# Epidemiology of psoriasis in hard-to-treat body locations: data from the Danish skin cohort

**DOI:** 10.1186/s12895-020-00099-7

**Published:** 2020-05-20

**Authors:** Alexander Egeberg, Kyoungah See, Alyssa Garrelts, Russel Burge

**Affiliations:** 1 Department of Dermatology and Allergy, Herlev and Gentofte Hospital, University of Copenhagen, Kildegårdsvej 28, 2900 Hellerup, Denmark; 2 Eli Lilly and Company, Indianapolis, Indiana, USA; 3 Division of Pharmaceutical Sciences, University of Cincinnati, Cincinnati, OH USA

**Keywords:** Psoriasis, Hard to treat, Genital, Nails, Epidemiology

## Abstract

**Background:**

Having psoriasis in hard-to-treat areas, i.e. the scalp, face, palms, soles, nails, and genitals, respectively, can impair patients’ quality of life. We investigated the prevalence of hard-to-treat body locations of psoriasis, and described patients’ clinical and demographic characteristics, and quality of life impacts in a population-based cohort.

**Methods:**

We performed a cross-sectional study using a total of 4016 adults (≥18 years) with psoriasis from the Danish Skin Cohort. Groups were compared to patients without involvement of hard-to-treat areas.

**Results:**

The most frequently affected hard-to-treat area was the scalp (43.0%), followed by the face (29.9%), nails (24.5%), soles (15.6%), genitals (14.1%), and palms (13.7%), respectively. Higher prevalence was generally seen with increasing psoriasis severity. Among all patients 64.8, 42.4, and 21.9% of patients had involvement of ≥1, ≥2, or ≥ 3 hard-to-treat areas. Those with involvement of certain hard-to-treat areas such as hands, feet, and genitals had clinically relevant DLQI impairments. Having involvement of one hard-to-treat area was significantly associated with other hard-to-treat areas affected even after adjusting for age, sex, and psoriasis severity.

**Conclusion:**

Psoriasis commonly affects hard-to-treat locations, even in patients with mild disease. For some of these areas, patient-reported disease burden, e.g. as measured by DLQI, is impaired.

## Background

Plaque psoriasis (henceforth “psoriasis”) is a common skin disease which affects approximately 2–3% of the world population [1], and as much as 8–11% of some Northern European countries [2, 3]. Psoriasis is associated with a number of comorbidities and greatly impacts patients’ health-related quality of life [4]. Involvement of the scalp, face, palms, soles, nails, and genitals can be particularly debilitating [5].

The scalp is a common site for psoriasis and the presence of hair can complicate the use of topical treatments [6], and the visibility and pruritus associated with scalp psoriasis may negatively impact patients’ quality of life [7]. Facial psoriasis was previously believed to be uncommon; however, high prevalence estimates have also been reported [8]. Palmoplantar psoriasis has a significant impact on quality of life and daily function, as measured by tools such as the Palmoplantar Quality Of Life Index [9–11]. Indeed, patients with palmoplantar involvement suffer from greater physical disability compared to those without palmoplantar involvement [12]. Although psoriasis limited only to the nails occurs in only 1 to 5% of patients [13], approximately 50% of psoriasis patients are affected by nail psoriasis at any given time [14, 15]. The lifetime incidence of nail disease in patients with psoriasis is estimated between 80 to 90% [16]. Between 29 and 63% of patients with psoriasis are impacted by psoriasis lesions in the genital area at some point during the course of the disease [17–21]. Collectively, these areas are considered to be hard-to-treat locations [22].

Despite the available research on patients with psoriasis in these hard-to-treat locations, the aforementioned studies were based on different study populations, and no studies have been conducted in a large, longitudinal cohort of patients simultaneously across all of these hard-to-treat locations. Furthermore, details on disease severity in terms of body surface area (BSA) and flares, as well as various symptom and quality of life impacts, are somewhat limited. The objectives of this study were to investigate the prevalence of hard-to-treat body locations of psoriasis, and to describe patients’ clinical and demographic characteristics, disease severity, psoriasis symptoms, and quality of life impacts in a population-based cohort.

## Methods

The study was approved by the Danish Data Protection Agency, and registered at the Capital Region’s inventory (Videnscenter for Dataanmeldelser, ref. VD-2018-286). This constitutes the necessary legal requirements. In Denmark, ethical reviews and informed consent is not required for observational studies not involving human tissue.

Data were drawn from the Danish Skin Cohort, a prospective cohort containing data on three groups of individuals (general population subjects, patients with psoriasis, and patients with atopic dermatitis, respectively). For this study, patients with a dermatologist verified diagnosis of plaque psoriasis were included (*n* = 4016). This study was a secondary analysis of existing data.

### Data collection

Data collection for the Danish Skin Cohort has been described in detail elsewhere [2]. Briefly, information on lifestyle and general health included height in cm, weight in kg, smoking history and quantity, and alcohol consumption. Information about disease activity included the number of flares in the past 12 months, and the percent currently affected body surface area (BSA). A flare was defined as one or more consecutive days with significant worsening of symptoms requiring escalation of treatment or seeking additional medical advice [23]. This definition was initially proposed for atopic dermatitis, but is presumed to work equally well for psoriasis. Quantitative measures of touch avoidance, skin [24] and joint pain, as well as pruritus [25], was recorded using a numeric rating scale (NRS) [26, 27], and patients were asked about the location of pruritus relative to psoriasis lesions. Among patients with genital psoriasis, patients were asked about the impact of their genital psoriasis on sexual activity and function using the Genital Psoriasis Sexual Impact Scale (GPSIS) [28]. Information on Dermatology Life Quality Index (DLQI) and EuroQoL 5 Dimensions 5 Levels (EQ-5D-5 L) was also recorded.

### Statistical analysis

Descriptive tables were generated for patients with psoriasis, with and without involvement of a hard-to-treat area, and subgroups based on disease severity. Mild psoriasis was defined as patients with a current BSA greater than 0 and less than 3, moderate psoriasis was defined as a BSA ≥3 and < 10, and patients with a BSA of ≥10 were considered to have severe psoriasis. Body mass index (BMI) was defined as weight in kg divided by the squared height in meters. The prevalence of each specific hard-to-treat location (as well as prevalence of having involvement of at least one, 2 or more, and 3 or more locations) were estimated using all psoriasis patients in the Danish Skin Cohort as reference. Between-group comparisons were made using patients without involvement of a hard-to-treat area as reference. Summary statistics were generated and expressed as mean and standard deviation (SD) for normally distributed variables, median and interquartile range (IQR) for non-normally distributed continuous variables and frequencies for categorical variables. Parametric variables were compared between groups using Student’s t-test, while Mann-Whitney U test was performed for non-parametric variables. Dichotomous variable comparisons are done using Pearson’s chi-square test. Odds ratios (ORs) with 95% confidence intervals (CIs) were calculated using logistic regression models. Dependent and independent variables, respectively, were the anatomical locations as outlines in Supplementary Figure 1. Variables included in the adjusted models (controlled variables: age, sex, and psoriasis severity) were selected a priori following consensus among all the study authors (and recorded in the internal statistical analysis plan) as these were deemed clinically relevant. All analyses were performed using STATA software version 13.0 (StataCorp, College Station, TX, USA).

## Results

From the Danish Skin Cohort, we identified 4016 patients with dermatologist verified plaque psoriasis. Of these, 2602 (64.8%) patients currently had psoriasis in at least one hard-to-treat location, whereas 1414 (35.2%) patients did not. Patients with psoriasis in hard-to-treat locations were slightly younger (57.8 vs 62.4 years), and with a female predominance among patients with involvement of the scalp, face, palms or feet, whereas genital or nail involvement we seen more often among men (Table 1 and supplementary Table 1). We observed significant differences in smoking habits in patients with all hard-to-treat areas except for those with genital psoriasis. With regards to BMI, age of psoriasis onset, current BSA, and number of flares per year, these findings were all significantly different when compared to patients without involvement of a hard-to-treat area.

**Table 1:** Overall characteristics of patients with psoriasis

	*With involvement of difficult-to-treat area*							*Without involvement of difficult-to-treat area*
	Scalp	Face	Palms	Soles	Genitals	Nails	At least one difficult-to-treat area	
	(*n* = 1726)	(*n* = 1200)	(*n* = 551)	(*n* = 628)	(*n* = 568)	(*n* = 982)	(*n* = 2602)	(*n* = 1414)
Age, mean (SD)	56.3 (15.1)	54.2 (14.9)	59.7 (13.4)	59.0 (12.9)	53.9 (14.3)	57.4 (13.4)	57.8 (14.4)	62.4 (13.9)
Sex, n (%)								
Women	924 (53.5)	620 (51.7)	347 (63.0)	412 (65.6)	237 (41.7)	477 (48.6)	1423 (54.7)	817 (57.8)
Men	802 (46.5)	580 (48.3)	204 (37.0)	216 (34.4)	331 (58.3)	505 (51.4)	1179 (45.3)	597 (42.2)
Smoking, n (%)								
Daily smoker	335 (19.4)	271 (22.6)	185 (33.6)	207 (33.0)	131 (23.1)	229 (23.3)	579 (22.2)	274 (19.4)
Occasional smoker	78 (4.5)	54 (4.5)	26 (4.7)	26 (4.1)	19 (3.4)	46 (4.7)	116 (4.5)	53 (3.8)
Former smoker	797 (46.2)	524 (43.7)	259 (47.0)	310 (39.4)	263 (46.3)	479 (48.8)	1221 (46.9)	638 (45.2)
Never smoker	512 (29.7)	350 (29.2)	79 (14.3)	82 (13.1)	152 (26.8)	225 (22.9)	679 (26.1)	433 (30.7)
Unknown	4 (0.2)	1 (0.1)	2 (0.4)	3 (0.5)	3 (0.5)	3 (0.3)	7 (0.3)	13 (0.9)
Units of alcohol per week, median (IQR)	2 (0–7)	2 (0–7)	2 (0–7)	2 (0–7)	2 (0–7)	2 (0–7)	2 (0–7)	3 (1–8)
Body mass index, n								
Underweight (BMI < 18.5)	26 (1.5)	18 (1.5)	13 (2.4)	12 (1.9)	8 (1.4)	14 (1.4)	47 (1.8)	30 (2.1)
Normal weight (BMI 18.5–25)	579 (33.6)	397 (33.1)	171 (31.0)	202 (32.2)	178 (31.3)	282 (28.7)	860 (33.1)	543 (38.4)
Overweight (BMI 25–30)	609 (35.3)	394 (32.8)	190 (34.5)	203 (32.3)	192 (33.8)	347 (35.3)	911 (35.0)	506 (35.8)
Moderately obese (BMI 30–35)	316 (18.3)	226 (18.8)	112 (20.3)	133 (21.2)	109 (19.2)	213 (21.7)	486 (18.7)	203 (14.4)
Severely obese (BMI 35–40)	110 (6.4)	93 (7.8)	44 (8.0)	50 (8.0)	45 (7.9)	78 (7.9)	178 (6.8)	69 (4.9)
Very severely/morbidly obese (BMI > 40)	7 (4.2)	60 (5.0)	18 (3.1)	21 (3.3)	30 (5.3)	41 (4.2)	99 (3.8)	30 (2.1)
Unknown	14 (0.8)	12 (1.0)	4 (0.7)	7 (1.1)	6 (1.1)	7 (0.7)	21 (0.8)	33 (2.3)
Age at psoriasis onset, mean (SD)	25.4 (16.9)	23.9 (16.0)	32.6 (17.8)	33.2 (17.7)	24.8 (15.9)	26.4 (16.4)	28.6 (17.7)	32.6 (18.9)
Current BSA, median (IQR)	5 (2–12)	5 (2–20)	5 (2–10)	4 (2–10)	5 (2–20)	5 (2–15)	4 (1–10)	1 (0–3)
Flares in last 12 months, n (%)								
None	379 (22.0)	196 (16.3)	87 (15.8)	116 (18.5)	79 (13.9)	171 (17.4)	584 (22.4)	434 (30.7)
1 flare	207 (12.0)	145 (12.1)	68 (12.3)	82 (13.1)	65 (11.4)	126 (12.8)	337 (13.0)	86 (6.1)
2–5 flares	584 (33.8)	445 (37.1)	166 (30.1)	194 (30.9)	204 (35.9)	343 (34.9)	862 (33.1)	158 (11.2)
6–10 flares	198 (11.5)	147 (12.3)	72 (13.1)	79 (12.6)	79 (13.9)	119 (12.1)	287 (11.0)	35 (2.5)
> 10 flares	278 (16.1)	213 (17.8)	135 (24.5)	131 (20.9)	111 (19.5)	181 (18.4)	419 (15.8)	46 (3.3)
Unknown	80 (4.6)	54 (4.5)	23 (4.2)	26 (4.1)	30 (5.3)	42 (4.3)	122 (4.7)	655 (46.3)

*BMI* Body mass index, *BSA* Body surface area, *IQR* Interquartile range, *SD* Standard deviation

1 unit of alcohol = 12 g of alcohol

### Prevalence of psoriasis in hard-to-treat areas

**Fig. 1:**
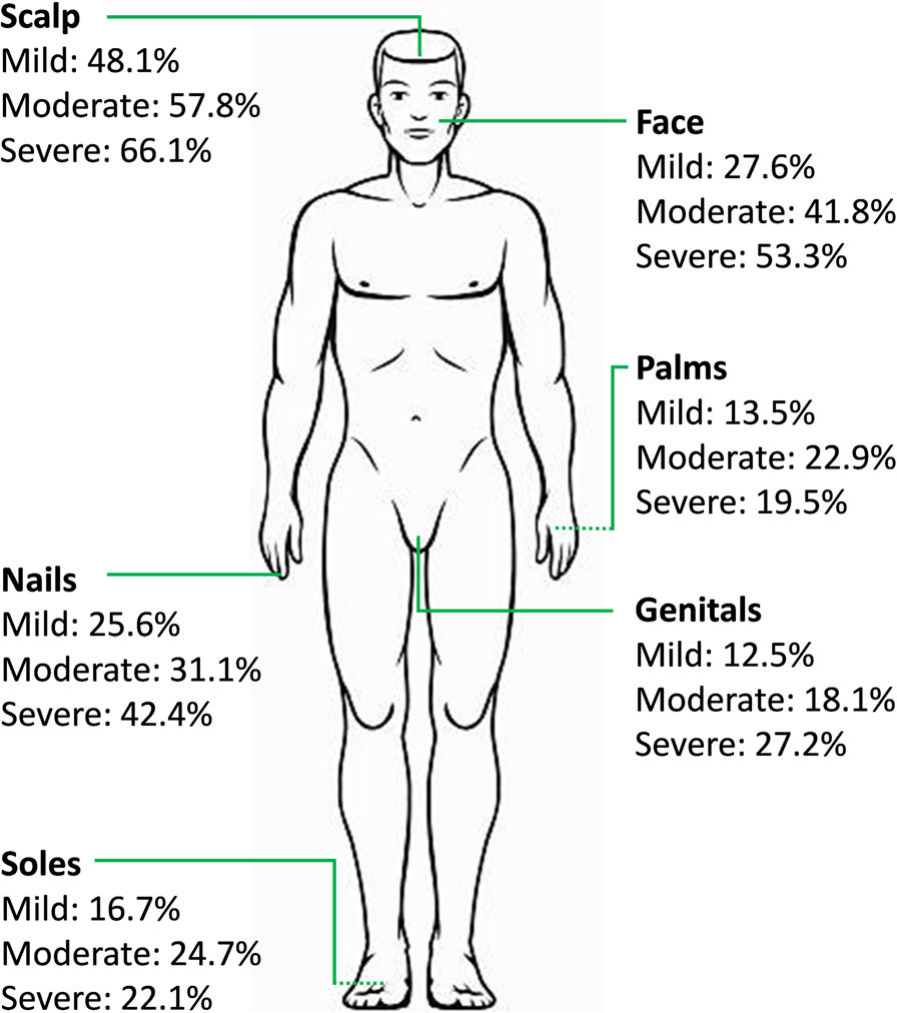
Prevalence of psoriasis in hard-to-treat areas across psoriasis severity

The most frequently affected hard-to-treat area was the scalp (43.0%; 1726/4016), followed by the face (29.9%; 1200/4016), nails (24.5%; 982/4016), soles (15.6%; 628/4016), genitals (14.1%; 568/4016), and palms (13.7%; 551/4016), respectively. Among all patients 64.8% (2602/4016), 42.4% (1702/4016), and 21.9% (878/4016) of patients had involvement of one or more, two or more, or three or more hard-to-treat areas. Stratified by disease severity (Fig. 1) prevalence of psoriasis in the scalp, face, genitals, and nails was increasing with increasing psoriasis severity. Affected palms and soles was most frequent among patients with moderate psoriasis (BSA ≥3 and < 10), likely due to the fact that the majority patients with palmoplantar involvement may have only these areas involved (i.e. 2 hands and 2 ft, which would correspond to a BSA of 4). Among patients with mild psoriasis, 80.4% of patients had involvement of at least one hard-to-treat area, whereas the prevalence was 89.0% among patients with severe psoriasis. Notably, 68.8 and 43.7% of patients with severe psoriasis had affected at least 2 and 3 hard-to-treat areas.

### Patient reported outcome measures in patients with psoriasis in hard-to-treat areas

**Fig. 2:**
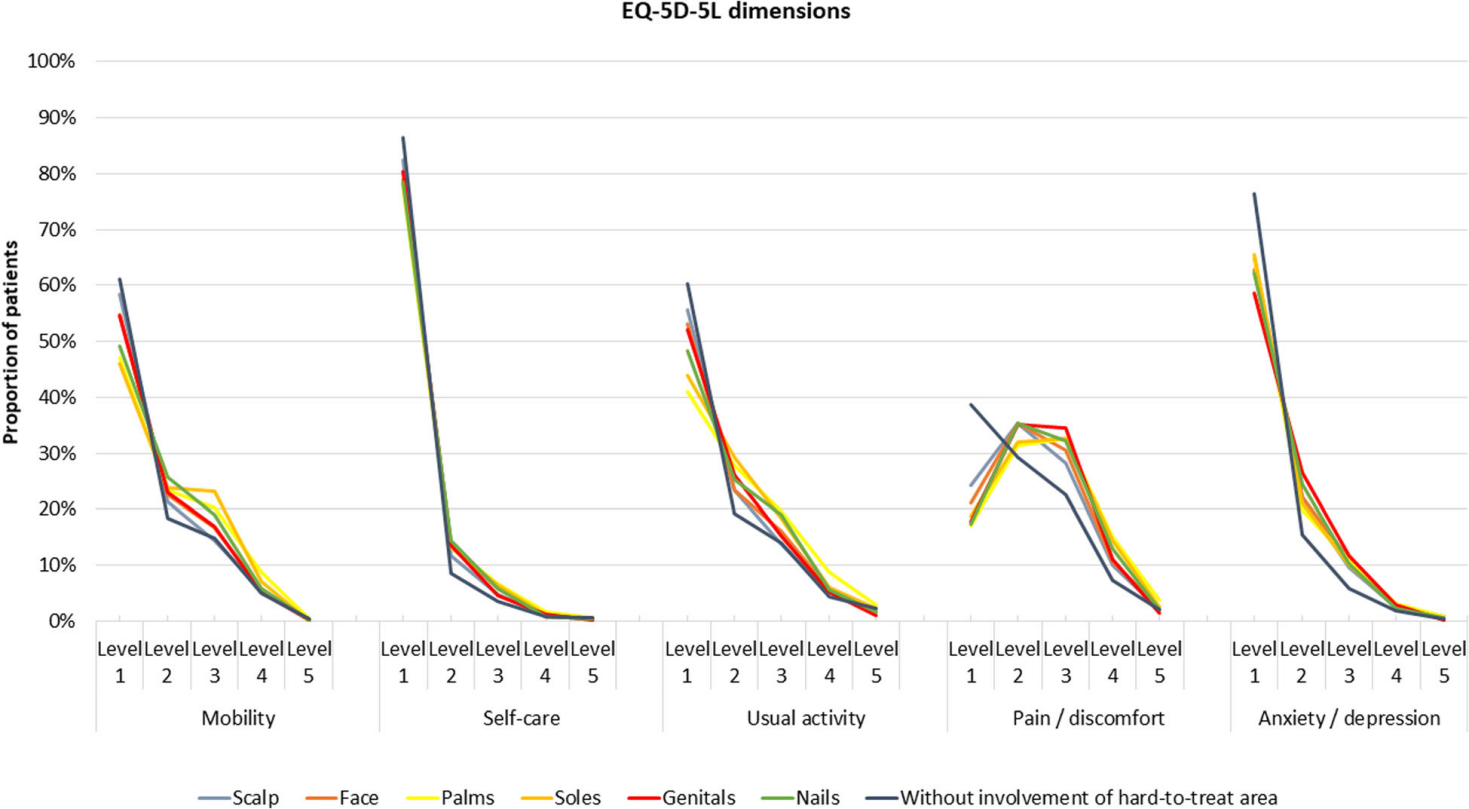
EQ-5D-5 L dimensions

EQ-5D-5 L data are shown in Fig. 2 and Supplementary Table 2. Compared with patients without involvement of a hard-to-treat area, all examined data points were differed to varying degrees. For example, 45.9, 47.1, and 49.1% of patients with affected soles, palms, or nails, respectively, reported no problems with mobility, compared with 61.2% of patients without hard-to-treat area involvement. Along those lines, between 9.6 and 10.6% of patients with psoriasis in hard-to-treat areas reported being moderately anxious or depressed, compared with 5.9% of patients without hard-to-treat area involvement (Supplementary Table 2). Among patients with psoriasis in hard-to-treat areas, 17.1–24.3% of patients reported no pain or discomfort, as opposed to 38.8% among those without psoriasis in hard-to-treat areas, respectively. Similarly, 10.0–15.1% vs. 7.3%, respectively, of patients reported severe pain or discomfort. Nonetheless, when asked to rate the level of skin pain on a NRS, this ranged from 2.5–3.5 among those with psoriasis in a hard-to-treat area vs. 1.2 for those without such involvement (Table 2). EQ-5D-VAS was generally lower for patients with psoriasis in a hard-to-treat area (Supplementary Table 2). The lowest DLQI was observed among patients without psoriasis in hard-to-treat areas (NRS 1.7), whereas the highest DLQI was seen among those with genital involvement (NRS 5.9), followed by palms and soles (both NRS 5.7), face and nails (both NRS 5.6), and scalp (NRS 4.7), respectively. The NRS for joint pain in patients without psoriasis in a hard-to-treat area was 3.5, and ranged from 3.9–4.7 among patients with involvements of such areas. Data restricted to people with mild disease are show in Supplementary 3.

**Table 2:** Impact of psoriasis in hard-to-treat areas

	*With involvement of difficult-to-treat area*							*Without involvement of difficult-to-treat area*
	Scalp	Face	Palms	Soles	Genitals	Nails	At least one difficult-to-treat area	
	(n = 1726)	(n = 1200)	(n = 551)	(n = 628)	(n = 568)	(n = 982)	(n = 2602)	(n = 1414)
	(mild = 561)	(mild = 322)	(mild = 157)	(mild = 195)	(mild = 146)	(mild = 299)	(mild = 937)	(mild = 229)
Current DLQI, mean (SD)	4.7 (5.2)	5.6 (5.5)	5.7 (5.8)	5.7 (5.9)	5.9 (5.6)	5.6 (5.2)	4.6 (5.1)	1.7 (3.4)
Current DLQI (mild psoriasis only), mean (SD)	2.4 (3.1)	3.0 (3.7)	3.5 (4.4)	3.2 (3.7)	3.2 (3.5)	3.0 (3.7)	2.6 (3.3)	1.6 (2.9)
Joint pain (NRS 0–10) in last 7 days, mean (SD)	3.9 (3.0)	4.1 (3.1)	4.7 (3.0)	4.7 (3.0)	4.0 (3.0)	4.4 (3.0)	4.0 (3.0)	3.5 (3.1)
Skin pain (NRS 0–10) in last 7 days, mean (SD)	2.5 (2.7)	2.8 (2.8)	3.5 (3.1)	3.4 (3.0)	3.0 (2.8)	3.0 (2.8)	2.5 (2.8)	1.2 (2.1)
Touch avoidance (NRS 0–10) in last 7 days, mean (SD)	1.2 (2.5)	1.5 (2.7)	1.8 (3.0)	1.4 (2.7)	1.7 (2.9)	1.5 (2.7)	1.2 (2.5)	0.4 (1.6)
Trouble sleeping (NRS 0–10) in last 3 days, mean (SD)	3.3 (3.0)	3.6 (3.0)	4.0 (3.1)	3.8 (3.1)	3.7 (3.0)	3.5 (3.0)	3.3 (3.0)	2.7 (2.9)
Itch severity (NRS 0–10) in last 3 days, mean (SD)	3.3 (2.9)	3.8 (3.0)	3.9 (3.2)	3.8 (3.0)	4.2 (3.0)	3.7 (2.9)	3.2 (2.9)	1.6 (2.3)
Itch location, n (%)								
Only on lesional skin	646 (37.4)	480 (40.0)	233 (42.3)	261 (41.6)	213 (37.5)	379 (38.6)	1031 (39.6)	270 (32.7)
Predominantly on lesional skin	492 (28.5)	374 (31.2)	156 (28.3)	172 (27.4)	189 (33.3)	303 (30.9)	664 (25.5)	103 (12.5)
Only on non-lesional skin	86 (5.0)	34 (2.8)	16 (2.9)	18 (2.9)	25 (4.4)	39 (4.0)	129 (5.0)	45 (5.4)
Predominantly on non-lesional skin	146 (8.5)	98 (8.2)	34 (6.2)	54 (8.6)	49 (8.6)	63 (6.4)	209 (8.0)	35 (4.2)
Equally on lesional and non-lesional skin	140 (8.1)	97 (8.1)	42 (7.6)	43 (6.9)	50 (8.8)	80 (8.2)	182 (7.0)	35 (4.2)
Patient generally does not have itch	212 (12.3)	15 (9.6)	68 (12.3)	79 (12.6)	42 (7.4)	115 (11.7)	381 (14.6)	331 (40.0)
Unknown	4 (0.2)	2 (0.2)	2 (0.4)	1 (0.2)	0 (0.0)	3 (0.3)	6 (0.2)	8 (1.0)

DLQI, dermatology life quality index; NRS, numerical rating scale; SD, standard deviation

### Sexual impact of genital psoriasis

Examination of the sexual impact of genital psoriasis (Supplementary Table 4), showed that 10% of patients with genital psoriasis reported that they had not been sexually active in the past week specifically due to their genital psoriasis, and with 9.9 and 12.7% of patients reporting that they “sometimes” or “often” avoided sexual activity due to their genital psoriasis, respectively. A low, moderate, high, and very high degree of worsening of genital psoriasis following sexual activity, respectively, was reported in 11.4, 11.1, 3.5, and 1.1% of patients with genital psoriasis (Supplementary Table 4). Similarly, almost one third of patients with genital psoriasis reported some degree of sexual impact in the DLQI questionnaire (question 9; Supplementary Table 5).

### Association between psoriasis in different hard-to-treat areas

In analyses adjusted for age, sex, and psoriasis severity (percent BSA currently affected), we observed significant associations between psoriasis in different hard-to-treat areas. For example, while the risk of having scalp psoriasis was 6-fold increased (OR 6.22, 95% CI 5.24–7.38) in patients with psoriasis in the face vs. those without facial involvement, the risk of having genital psoriasis (OR 3.98, 95% CI 3.24–4.88) and nail psoriasis (OR 2.78, 95% CI 2.36–3.27) was also significantly increased (Supplementary Figure 1 and Supplementary Table 6). Similarly, there was 10-fold increased risk of having palmar involvement among patients with psoriasis on the soles of their feet (OR 10.09, 95% CI 8.19–12.42), and these patients also had a 91% increased risk of nail involvement (OR 1.91, 95% CI 1.59–2.31).

### Multiple comparisons

Due to the large number of analyses, we performed post-hoc analyses where *p*-values were corrected according to the Benjamini-Hochberg procedure. Results from these analyses yielded similar findings compared with our main analyses, and the Benjamini-Hochberg p-values are listed in Supplementary Table 7.

## Discussion

In this cross-sectional study of Danish patients with plaque psoriasis, the prevalence of psoriasis in hard-to-treat areas was high. Notably, burden of disease and quality of life impairments was greater among patients with psoriasis in hard-to-treat locations compared with patients without involvement of hard-to-treat areas, although the absolute differences ranged depending on the outcome. Data on the minimum clinically important differences (MCID) for many of these outcomes are lacking, but where such data are available far from every outcome in our study met the MCID among patients with involvement of a hard-to-treat area. Patients with psoriasis in a hard-to-treat area had significantly increased risk of having psoriasis in other hard-to-treat areas, even after adjustment for potential confounders such as psoriasis severity.

The prevalence of psoriasis in hard-to-treat areas differs greatly across studies. For example, one French study [29] of 776 patients with psoriasis from two tertiary centres found that 43.2% of patients currently had genital psoriasis. This prevalence is somewhat higher compared to the findings of our and previous studies. For example, one study from India found a genital psoriasis prevalence of 11.7% [30], whereas a Dutch study reported that 29% of patients had genital involvement [31]. In a study of 2009 German patients with psoriasis [5], 16.5% of patients had genital psoriasis. Despite these somewhat wide variations, genital involvement appears to be quite common among patients with psoriasis, and as demonstrated by our study, genital psoriasis may have a considerable impact on patients’ sexual activity and function, and consequently on their quality of life.

In agreement with our results, Larsabal et al. [29] found positive associations between genital psoriasis and nail psoriasis (OR 1.9, 95% CI 1.3–2.8) and scalp psoriasis (OR 1.9, 95% CI 1.3–2.6). However, that study also reported a slight inverse association between genital psoriasis and palmoplantar psoriasis (OR 0.5, 95% CI 0.3–0.9) whereas we found no association between genital psoriasis and psoriasis on the palms or soles. Although the reason for this difference is unclear, the study by Larsabal and colleagues [29] was not limited to plaque psoriasis but also included e.g. pustular psoriasis which may potentially explain the inverse association.

In line with our study, Augustin and colleagues reported that 65.4% of patients had scalp psoriasis, whereas 44.8% of patients had facial involvement [5]. However, while our study to a large degree is in agreement with previous studies, our findings expand the existing literature considerably by providing severity-specific estimates in a cohort of more than four thousand patients with psoriasis.

We found significant reductions in patient reported outcome measures among patients with psoriasis in hard-to-treat areas, suggesting that these patients may comprise the populations with the greatest disease burden among patients with psoriasis. Nonetheless, appropriate context is important when interpreting reductions in patient reported coutcomes. In the literature, the MCID for the Itch NRS has been reported to be 2–3, and it has been proposed that the MCID for DLQI in patients with inflammatory skin disorders should be 4 [32, 33]. As there was more than a 4-point difference among DLQI for hands, feet, and genitals, this would indeed be considered clincially relevant. While the MCID for postoperative pain was previously reported to be 18.6–22.6 mm on a 100 mm VAS (i.e. roughly corresponding to 2 on our NRS) [34], post-operative pain is arguably different from skin or joint pain resulting from a chronic dermatologic condition, and as we are unaware of any published data on MCID for skin pain in psoriasis or AD, this may hinder a thorough interpretation of the clinical relevance of the observed differences in our study. With a MCID of 3 for itch, the observed differences in our study would not be considered clinically relevant, as itch in most of these hard-to-treat areas differed by greater than 2 but less than 3. Intestingly, approximately one in 10 patients reported that their itch was only or predominantly located to non-lesional skin. Even more noticeable however, was the fact that among patients without involvement of a hard-to-treat area, 40% reported that they did not itch (currently, or in general), whereas this only was reported for 7.4–12.6% of patients with psoriasis in a hard-to-treat area did not itch. Notably however, many of the patient reported outcomes were attenuated when limited to patients with mild disease, albeit the impact of several hard-to-treat area involvement remained apparent for many outcomes. To date, many guidelines focus predominantly on quantitative scores such as BSA or Psoriasis Area and Severity Index, and although DLQI to a lesser extent may be included in some recommendations, use of BSA or PASI may signficantly underestimate the disease impact among patients with relatively limited disease located to hard-to-treat areas such as the genitals. Indeed, our findings expand the current litterature considerably by thoroughly describing the burden and patient-perceived impact of psoriasis in hard-to-treat areas. These findings may support clinicians to identify patients that, despite having less widespread disease, would have particular benefit of intensified treatment of their psoriasis. Notably, novel therapies such as biologics appear to have beneficial effects on depressive symptoms, which may be of particular relevance in patients with high subjective disease impact, such as those with e.g. genital psoriasis [35]. Importantly however, several barriers to treatment with biologics have been reported, including cost, reimbursement and fear of recourse [36]. On the other hand, patients generally report greater satisfaction and efficacy, and, in turn adherance to treatment with biologics compared with other systemic agents [37].

Certain limitations and strengths warrants mentioning. Although we had available data on psoriasis severity, including the percentage of affected BSA, we did not have data on the extent of psoriasis in individual hard-to-treat areas, e.g. whether patients had one small plaque in their scalp or whether the entire scalp was covered with psoriasis lesions. It is likely that such data would have provided even more granularity and aided interpretation of the study results. Our study was strengthened by the sheer number of patients, and the detailed information on itch severity and location, as well as data on sexual impact and function, which may provide further insight into particularly vulnerable subgroups of patients with psoriasis.

## Conclusion

We found that psoriasis commonly affects hard-to-treat locations, even in patients with mild disease. These novel findings highlight unmet treatment needs that persist among patients with psoriasis, suggesting a considerable potential for optimization of current treatment approaches not only among severe psoriasis, but also in patients with mild or moderate disease. Importantly however, while patients with psoriasis in hard-to-treat locations scored poorer on many patient reported outcome measures in our study, only some of these, e.g. DLQI responses, translated into a clincially relevant difference compared to patients without involvement of a hard-to-treat area.

## Supplementary information

**Additional file 1: Figure S1** Associations between different hard-to-treat locations. **Table S1** Tests for significant differences. **Table S2** EQ-5D-5 L. **Table S3** Impact of psoriasis in hard-to-treat areas among patients with mild disease (BSA < 3). **Table S4** Genital Psoriasis Sexual Impact Scale. **Table S5** DLQI question 9 in patients with genital psoriasis. **Table S6** Adjusted odds ratios with 95% confidence intervals of association between different hard-to-treat areas. **Table S7***P*-values for all estimates, adjusted using the Benjamini-Hochberg procedure. **Table S8** Distribution of psoriasis severity and affection of a hard-to-treat area

## Data Availability

The datasets generated and/or analyzed during the current study are not publicly available due to national security requirements but are available from the corresponding author following requestors approval from the Danish Data Agency and Statistics Denmark.

## Abbreviations

BMI: Body mass index
BSA: Body Surface Area
CI: Confidence interval
DLQI: Dermatology life quality index
EQ-5D-5 L: EuroQoL 5 Dimensions 5 Levels
GPSIS: Genital Psoriasis Sexual Impact Scale
IQR: Interquartile range
MCID: Minimum clinically important differences
NRS: Numerical rating scale
OR: Odds ratio
SD: Standard deviation
