# Supplementary material for: Epidemiology of psoriasis in hard-to-treat body locations: data from the Danish skin cohort
Source: BMC Dermatol. 2020 May 20;20:3. doi: 10.1186/s12895-020-00099-7 (PMC7238562; doi:10.1186/s12895-020-00099-7)
Supplement: Supplementary file 1 — Additional file 1: Figure S1 Associations between different hard-to-treat locations. Table S1 Tests for significant differences. Table S2 EQ-5D-5 L. Table S3 Impact of psoriasis in hard-to-treat areas among patients with mild disease (BSA < 3). Table S4 Genital Psoriasis Sexual Impact Scale. Table S5 DLQI question 9 in patients with genital psoriasis. Table S6 Adjusted odds ratios with 95% confidence intervals of association between different hard-to-treat areas. Table S7P-values for all estimates, adjusted using the Benjamini-Hochberg procedure. Table S8 Distribution of psoriasis severity and affection of a hard-to-treat area [file 12895_2020_99_MOESM1_ESM.docx]

**Supplementary Figure 1 – Associations between different hard-to-treat locations**

The figure shows the associations (presented as odds ratios with 95% confidence intervals) between psoriasis in hard-to-treat areas. Estimates are adjusted for age, sex, and psoriasis severity.

**Supplementary table 1 –Tests for significant differences**

| **Differences compared to patients without involvement of a hard-to-treat area in manuscript table 1** | | | | |
| --- | --- | --- | --- | --- |
|  |  |  |  |  |
|  | **p<0.05** | **p<0.01** | **p<0.001** | **p<0.0001** |
| Age |  |  |  | Scalp, Face, Palms, Soles, Genitals, Nails |
| Sex | Face | Palms |  | Soles, Genitals, Nails |
| Smoking | Face |  |  | Palms, Soles, Nails |
| Alcohol |  | Genitals, Nails | Scalp | Face, Palms, Soles |
| BMI |  |  |  | Scalp, Face, Genitals, Nails |
| Age of onset |  |  |  | Scalp, Face, Genitals, Nails |
| Current BSA |  |  |  | Scalp, Face, Palms, Soles, Genitals, Nails |
| Flares |  |  |  | Scalp, Face, Nails |

**Differences compared to patients without involvement of a hard-to-treat area in manuscript table 2**

| \|  \| **p<0.05** \| **p<0.01** \| **p<0.001** \| **p<0.0001** \| \| --- \| --- \| --- \| --- \| --- \| \| DLQI \|  \|  \|  \| Scalp, Face, Palms, Soles, Genitals, Nails \| \| Joint pain \|  \| Genitals \| Scalp \| Face, Palms, Soles, Nails \| \| Skin Pain \|  \|  \|  \| Scalp, Face, Palms, Soles, Genitals, Nails \| \| Touch avoidance \|  \|  \|  \| Scalp, Face, Palms, Soles, Genitals, Nails \| \| Trouble sleeping \|  \|  \|  \| Scalp, Face, Palms, Soles, Genitals, Nails \| \| Itch severity \|  \|  \|  \| Scalp, Face, Palms, Soles, Genitals, Nails \| \| Itch location \|  \|  \|  \| Scalp, Face, Palms, Soles, Genitals, Nails \| |
| --- | --- | --- | --- | --- | --- | --- | --- | --- | --- | --- | --- | --- | --- | --- | --- | --- | --- | --- | --- | --- | --- | --- | --- | --- | --- | --- | --- | --- | --- | --- | --- | --- | --- | --- | --- | --- | --- | --- | --- | --- |

**Supplementary table 2 – EQ-5D-5L**

|  |  | **Plaque psoriasis** | | | | | | | |
| --- | --- | --- | --- | --- | --- | --- | --- | --- | --- |
|  |  | ***With involvement of hard-to-treat area*** | | | | | |  | ***Without involvement of hard-to-treat area*** |
| **EQ-5D dimension** | | Scalp | Face | Palms | Soles | Genitals | Nails |  |  |
|  |  |  |  |  |  |  |  |  |  |
| **Mobility** | Level 1 | 58.3% | 54.8% | 47.1% | 45.9% | 54.6% | 49.1% |  | 61.2% |
|  | Level 2 | 21.3% | 22.7% | 23.5% | 23.8% | 23.1% | 25.7% |  | 18.5% |
|  | Level 3 | 14.5% | 16.7% | 20.4% | 23.1% | 16.9% | 19.1% |  | 14.9% |
|  | Level 4 | 5.6% | 5.7% | 8.7% | 7.2% | 5.3% | 5.9% |  | 5.1% |
|  | Level 5 | 0.3% | 0.3% | 0.4% | 0.0% | 0.2% | 0.2% |  | 0.3% |
|  |  |  |  |  |  |  |  |  |  |
| **Self-care** | Level 1 | 82.4% | 79.0% | 77.7% | 78.7% | 80.5% | 78.4% |  | 86.5% |
|  | Level 2 | 11.6% | 13.8% | 13.4% | 13.2% | 13.6% | 14.5% |  | 8.6% |
|  | Level 3 | 4.9% | 5.9% | 6.7% | 6.5% | 4.6% | 5.9% |  | 3.5% |
|  | Level 4 | 0.8% | 0.8% | 1.6% | 1.1% | 1.2% | 0.8% |  | 0.8% |
|  | Level 5 | 0.4% | 0.4% | 0.5% | 0.5% | 0.2% | 0.4% |  | 0.6% |
|  |  |  |  |  |  |  |  |  |  |
| **Usual activity** | Level 1 | 55.6% | 53.2% | 41.0% | 44.0% | 52.1% | 48.4% |  | 60.2% |
|  | Level 2 | 23.4% | 23.5% | 27.8% | 29.3% | 26.2% | 25.3% |  | 19.2% |
|  | Level 3 | 13.9% | 16.1% | 19.6% | 18.5% | 15.3% | 18.9% |  | 14.1% |
|  | Level 4 | 5.2% | 5.6% | 8.7% | 6.1% | 5.3% | 5.7% |  | 4.3% |
|  | Level 5 | 2.0% | 1.7% | 2.9% | 2.2% | 1.1% | 1.7% |  | 2.2% |
|  |  |  |  |  |  |  |  |  |  |
| **Pain / discomfort** | Level 1 | 24.3% | 21.2% | 17.1% | 18.8% | 17.8% | 17.3% |  | 38.8% |
|  | Level 2 | 35.4% | 35.4% | 31.5% | 32.1% | 35.2% | 35.4% |  | 29.3% |
|  | Level 3 | 28.3% | 30.4% | 32.7% | 32.5% | 34.5% | 32.3% |  | 22.6% |
|  | Level 4 | 10.0% | 10.6% | 15.1% | 14.4% | 11.1% | 13.0% |  | 7.3% |
|  | Level 5 | 2.0% | 2.4% | 3.6% | 2.6% | 1.4% | 2.0% |  | 2.1% |
|  |  |  |  |  |  |  |  |  |  |
| **Anxiety / depression** | Level 1 | 64.9% | 62.9% | 65.0% | 65.6% | 58.6% | 62.2% |  | 76.3% |
|  | Level 2 | 22.2% | 22.2% | 19.9% | 21.2% | 26.6% | 24.5% |  | 15.5% |
|  | Level 3 | 9.6% | 11.0% | 11.1% | 10.1% | 11.6% | 10.3% |  | 5.9% |
|  | Level 4 | 2.5% | 3.0% | 3.1% | 2.7% | 3.0% | 2.4% |  | 1.9% |
|  | Level 5 | 0.8% | 0.8% | 0.9% | 0.5% | 0.2% | 0.6% |  | 0.4% |
|  |  |  |  |  |  |  |  |  |  |
|  |  |  |  |  |  |  |  |  |  |
|  | **EQ-5D-VAS** |  |  |  |  |  |  |  |  |
|  |  |  |  |  |  |  |  |  |  |
|  | Mean | 65.8 | 64.7 | 63.2 | 62.7 | 64.9 | 63.4 |  | 69.1 |
|  | SD | 26.3 | 25.5 | 26.4 | 25.8 | 24.7 | 25.7 |  | 27.8 |
|  |  |  |  |  |  |  |  |  |  |
|  | Median | 75 | 70 | 70 | 70 | 70 | 70 |  | 80 |
|  | 25th | 50 | 50 | 50 | 50 | 50 | 50 |  | 50 |
|  | 75th | 85 | 85 | 85 | 80 | 84 | 80 |  | 90 |
|  |  |  |  |  |  |  |  |  |  |
|  |  |  |  |  |  |  |  |  |  |

All results are significantly different (p<0.001) compared with patients without involvement of a hard-to-treat area.

Note: Mobility, Self-Care, and Usual Activities domains include levels: 1="no problems", 2="slight problems", 3="moderate problems", 4="severe problems", 5="unable". The Pain/Discomfort domain includes levels: 1="no pain or discomfort", 2="slight pain or discomfort", 3="moderate pain or discomfort", 4="severe pain or discomfort", 5="extreme pain or discomfort". The Anxiety/Depression domain includes levels: 1="not anxious or depressed", 2="slightly anxious or depressed", 3="moderately anxious or depressed", 4="severely anxious or depressed", 5="extremely anxious or depressed".

**Supplementary Table 3 – Impact of psoriasis in hard-to-treat areas among patients with mild disease (BSA<3)**

|  | *With involvement of difficult-to-treat area* | | | | | |  | *Without involvement of difficult-to-treat area* | |
| --- | --- | --- | --- | --- | --- | --- | --- | --- | --- |
|  | Scalp | Face | Palms | Soles | Genitals | Nails |  | |  |
|  |  |  |  |  |  |  |  | |  |
|  |  |  |  |  |  |  |  | |  |
| Current DLQI, mean (SD) | 2.4 (3.1) | 3.0 (3.7) | 3.5 (4.4) | 3.2 (3.7) | 3.2 (3.5) | 3.0 (3.7) |  | | 1.6 (2.9) |
|  |  |  |  |  |  |  |  | |  |
| Joint pain (NRS 0-10) in last 7 days, mean (SD) | 3.4 (2.9) | 3.6 (2.9) | 4.0 (2.9) | 4.0 (2.9) | 3.2 (2.8) | 3.8 (2.9) |  | | 3.5 (2.9) |
|  |  |  |  |  |  |  |  | |  |
| Skin pain (NRS 0-10) in last 7 days, mean (SD) | 1.5 (2.1) | 1.9 (2.3) | 2.2 (2.5) | 2.4 (2.3) | 1.8 (2.1) | 1.9 (2.2) |  | | 1.6 (2.1) |
|  |  |  |  |  |  |  |  | |  |
| Touch avoidance (NRS 0-10) in last 7 days, mean (SD) | 0.5 (1.6) | 0.7 (1.9) | 1.1 (2.3) | 0.5 (1.4) | 0.8 (2.1) | 0.7 (1.9) |  | | 0.5 (1.6) |
|  |  |  |  |  |  |  |  | |  |
| Trouble sleeping (NRS 0-10) in last 3 days, mean (SD) | 2.8 (2.9) | 3.3 (2.9) | 3.2 (2.9) | 2.9 (2.8) | 3.2 (2.9) | 2.9 (2.8) |  | | 2.8 (2.8) |
|  |  |  |  |  |  |  |  | |  |
| Itch severity (NRS 0-10) in last 3 days, mean (SD) | 2.0 (2.2) | 2.3 (2.3) | 2.5 (2.6) | 2.7 (2.4) | 2.6 (2.4) | 2.3 (2.3) |  | | 2.1 (2.3) |
|  |  |  |  |  |  |  |  | |  |
| Itch location, n (%) |  |  |  |  |  |  |  | |  |
| Only on lesional skin | 224 (39.9) | 140 (43.5) | 78 (49.7) | 92 (47.2) | 66 (45.2) | 119 (39.8) |  | | 83 (36.2) |
| Predominantly on lesional skin | 105 (18.7) | 71 (22.1) | 30 (19.1) | 35 (18.0) | 31 (21.2) | 65 (21.7) |  | | 23 (10.0) |
| Only on non-lesional skin | 40 (7.1) | 14 (4.4) | 4 (2.6) | 10 (5.1) | 11 (7.5) | 17 (5.7) |  | | 11 (4.8) |
| Predominantly on non-lesional skin | 55 (9.8) | 26 (8.1) | 4 (2.6) | 15 (7.7) | 15 (10.3) | 22 (7.4) |  | | 13 (5.7) |
| Equally on lesional and non-lesional skin | 28 (5.0) | 18 (5.6) | 6 (3.8) | 7 (3.6) | 7 (4.8) | 13 (4.4) |  | | 12 (5.2) |
| Patient generally does not have itch | 109 (19.4) | 52 (16.2) | 35 (22.3) | 36 (18.5) | 16 (11.0) | 63 (21.1) |  | | 86 (37.6) |
| Unknown | 0 (0.0) | 1 (0.3) | 0 (0.0) | 0 (0.0) | 0 (0.0) | 0 (0.0) |  | | 1 (0.4) |
|  |  |  |  |  |  |  |  | |  |
| DLQI, dermatology life quality index; NRS, numerical rating scale; SD, standard deviation | | | |  |  |  |  | |  |

**Supplementary Table 4 – Genital Psoriasis Sexual Impact Scale**

|  |  | **Genital psoriasis** |
| --- | --- | --- |
|  |  | (n=568) |
|  |  |  |
| Question 1 | **In the past week, were you sexually active?** |  |
|  | Yes | 288 (50.7) |
|  | No, due to my genital psoriasis | 57 (10.0) |
|  | No, due to reasons other than my genital psoriasis | 218 (38.4) |
|  | Missing | 5 (0.9) |
|  |  |  |
|  | **In the past week, how often did you avoid sexual activity because of your genital psoriasis symptoms?** |  |
| Question 2 | Never | 203 (70.5) |
|  | Rarely | 34 (11.8) |
|  | Sometimes | 38 (13.2) |
|  | Often | 13 (4.5) |
|  | Missing | 0 (0.0) |
|  |  |  |
|  | **In the past week, how would you rate the level (degree) of worsening of your genital psoriasis symptoms during or following sexual activity?** |  |
| Question 3 | Very high | 1 (0.4) |
|  | High | 7 (2.4) |
|  | Moderate | 36 (12.5) |
|  | Low | 43 (14.9) |
|  | Very low of none at all | 201 (69.8) |
|  | Missing | 0 (0.0) |
|  |  |  |

Results in questions 2 and 3 are presented for patients answering “yes” in question 1.

**Supplementary Table 5 – DLQI question 9 in patients with genital psoriasis**

| **DLQI question 9** | |
| --- | --- |
| Over the last week, how much has your skin caused any sexual difficulties? | |
|  |  |
|  | **Genital psoriasis, n (%)** |
|  |  |
| Very much | 17 (3.0) |
| A lot | 45 (7.9) |
| A little | 99 (17.4) |
| Not at all | 285 (50.2) |
| Not applicable | 119 (21.0) |
| Missing | 3 (0.5) |

**Supplementary Table 6 – Adjusted odds ratios with 95% confidence intervals of association between different hard-to-treat areas**

|  | **OR** | **Lower CI** | **Upper CI** | **p-value** |
| --- | --- | --- | --- | --- |
| **Scalp** |  |  |  |  |
| Face | 6.21 | 5.23 | 7.36 | 0.0001 |
| Palms | 0.49 | 0.40 | 0.59 | 0.0001 |
| Soles | 0.49 | 0.41 | 0.59 | 0.0001 |
| Genitals | 2.81 | 2.27 | 3.47 | 0.0001 |
| Nails | 2.14 | 1.83 | 2.52 | 0.0001 |
|  |  |  |  |  |
| **Face** |  |  |  |  |
| Scalp | 6.22 | 5.24 | 7.38 | 0.0001 |
| Palms | 0.68 | 0.55 | 0.84 | 0.0004 |
| Soles | 0.67 | 0.55 | 0.82 | 0.0001 |
| Genitals | 3.98 | 3.24 | 4.88 | 0.0001 |
| Nails | 2.78 | 2.36 | 3.27 | 0.0001 |
|  |  |  |  |  |
| **Palms** |  |  |  |  |
| Face | 0.68 | 0.55 | 0.84 | 0.0003 |
| Scalp | 0.49 | 0.40 | 0.60 | 0.0001 |
| Soles | 10.10 | 8.20 | 12.43 | 0.0001 |
| Genitals | 0.96 | 0.74 | 1.24 | 0.7354 |
| Nails | 1.63 | 1.34 | 1.99 | 0.0001 |
|  |  |  |  |  |
| **Soles** |  |  |  |  |
| Face | 0.66 | 0.54 | 0.81 | 0.0001 |
| Scalp | 0.49 | 0.41 | 0.59 | 0.0001 |
| Palms | 10.09 | 8.19 | 12.42 | 0.0001 |
| Genitals | 1.11 | 0.87 | 1.41 | 0.4119 |
| Nails | 1.91 | 1.59 | 2.31 | 0.0001 |
|  |  |  |  |  |
| **Genitals** |  |  |  |  |
| Face | 4.03 | 3.29 | 4.94 | 0.0001 |
| Scalp | 2.86 | 2.31 | 3.53 | 0.0001 |
| Palms | 0.96 | 0.74 | 1.24 | 0.7501 |
| Soles | 1.11 | 0.87 | 1.42 | 0.3883 |
| Nails | 2.93 | 2.42 | 3.56 | 0.0001 |
|  |  |  |  |  |
| **Nails** |  |  |  |  |
| Face | 2.78 | 2.36 | 3.27 | 0.0001 |
| Scalp | 2.16 | 1.84 | 2.54 | 0.0001 |
| Palms | 1.63 | 1.34 | 1.99 | 0.0001 |
| Soles | 1.91 | 1.59 | 2.31 | 0.0001 |
| Genitals | 2.88 | 2.38 | 3.51 | 0.0001 |
|  |  |  |  |  |
| CI, confidence interval; OR, odds ratio | | |  |  |

Estimates are adjusted for age, sex, and psoriasis severity (%BSA)

**Supplementary Table 7 – P-values for all estimates, adjusted using the Benjamini-Hochberg procedure**

| **Label** | **Benjamini-Hochberg significance** | **Benjamini-Hochberg P-value** |
| --- | --- | --- |
| (Table 1) Scalp, DLQI | significant | <0.001 |
| (Table 1) Scalp, Joint pain | significant | <0.001 |
| (Table 1) Scalp, Skin Pain | significant | <0.001 |
| (Table 1) Scalp, Touch avoidance | significant | <0.001 |
| (Table 1) Scalp, Trouble sleeping | significant | <0.001 |
| (Table 1) Scalp, Itch severity | significant | <0.001 |
| (Table 1) Scalp, Itch location | significant | <0.001 |
| (Table 1) Face, DLQI | significant | <0.001 |
| (Table 1) Face, Joint pain | significant | <0.001 |
| (Table 1) Face, Skin Pain | significant | <0.001 |
| (Table 1) Face, Touch avoidance | significant | <0.001 |
| (Table 1) Face, Trouble sleeping | significant | <0.001 |
| (Table 1) Face, Itch severity | significant | <0.001 |
| (Table 1) Face, Itch location | significant | <0.001 |
| (Table 1) Palms, DLQI | significant | <0.001 |
| (Table 1) Palms, Joint pain | significant | <0.001 |
| (Table 1) Palms, Skin Pain | significant | <0.001 |
| (Table 1) Palms, Touch avoidance | significant | <0.001 |
| (Table 1) Palms, Trouble sleeping | significant | <0.001 |
| (Table 1) Palms, Itch severity | significant | <0.001 |
| (Table 1) Palms, Itch location | significant | <0.001 |
| (Table 1) Soles, DLQI | significant | <0.001 |
| (Table 1) Soles, Joint pain | significant | <0.001 |
| (Table 1) Soles, Skin Pain | significant | <0.001 |
| (Table 1) Soles, Touch avoidance | significant | <0.001 |
| (Table 1) Soles, Trouble sleeping | significant | <0.001 |
| (Table 1) Soles, Itch severity | significant | <0.001 |
| (Table 1) Soles, Itch location | significant | <0.001 |
| (Table 1) Genitals, DLQI | significant | <0.001 |
| (Table 1) Genitals, Joint pain | significant | 0.001 |
| (Table 1) Genitals, Skin Pain | significant | <0.001 |
| (Table 1) Genitals, Touch avoidance | significant | <0.001 |
| (Table 1) Genitals, Trouble sleeping | significant | <0.001 |
| (Table 1) Genitals, Itch severity | significant | <0.001 |
| (Table 1) Genitals, Itch location | significant | <0.001 |
| (Table 1) Nails, DLQI | significant | <0.001 |
| (Table 1) Nails, Joint pain | significant | <0.001 |
| (Table 1) Nails, Skin Pain | significant | <0.001 |
| (Table 1) Nails, Touch avoidance | significant | <0.001 |
| (Table 1) Nails, Trouble sleeping | significant | <0.001 |
| (Table 1) Nails, Itch severity | significant | <0.001 |
| (Table 1) Nails, Itch location | significant | <0.001 |
| (Table 2) Scalp, Age | significant | <0.001 |
| (Table 2) Scalp, Sex | significant | <0.001 |
| (Table 2) Scalp, Smoking | significant | <0.001 |
| (Table 2) Scalp, Alcohol | significant | 0.000 |
| (Table 2) Scalp, BMI | significant | <0.001 |
| (Table 2) Scalp, Age of onset | significant | <0.001 |
| (Table 2) Scalp, Current BSA | significant | <0.001 |
| (Table 2) Scalp, Flares | significant | <0.001 |
| (Table 2) Face, Age | significant | <0.001 |
| (Table 2) Face, Sex | significant | 0.002 |
| (Table 2) Face, Smoking | significant | 0.010 |
| (Table 2) Face, Alcohol | significant | <0.001 |
| (Table 2) Face, BMI | significant | <0.001 |
| (Table 2) Face, Age of onset | significant | <0.001 |
| (Table 2) Face, Current BSA | significant | <0.001 |
| (Table 2) Face, Flares | significant | <0.001 |
| (Table 2) Palms, Age | significant | <0.001 |
| (Table 2) Palms, Sex | significant | 0.036 |
| (Table 2) Palms, Smoking | significant | <0.001 |
| (Table 2) Palms, Alcohol | significant | <0.001 |
| (Table 2) Palms, BMI | significant | <0.001 |
| (Table 2) Palms, Age of onset | significant | <0.001 |
| (Table 2) Palms, Current BSA | significant | <0.001 |
| (Table 2) Palms, Flares | significant | <0.001 |
| (Table 2) Soles, Age | significant | <0.001 |
| (Table 2) Soles, Sex | significant | <0.001 |
| (Table 2) Soles, Smoking | significant | <0.001 |
| (Table 2) Soles, Alcohol | significant | <0.001 |
| (Table 2) Soles, BMI | significant | <0.001 |
| (Table 2) Soles, Age of onset | significant | <0.001 |
| (Table 2) Soles, Current BSA | significant | <0.001 |
| (Table 2) Soles, Flares | significant | <0.001 |
| (Table 2) Genitals, Age | significant | <0.001 |
| (Table 2) Genitals, Sex | significant | <0.001 |
| (Table 2) Genitals, Smoking | significant | <0.001 |
| (Table 2) Genitals, Alcohol | significant | 0.003 |
| (Table 2) Genitals, BMI | significant | <0.001 |
| (Table 2) Genitals, Age of onset | significant | <0.001 |
| (Table 2) Genitals, Current BSA | significant | <0.001 |
| (Table 2) Genitals, Flares | significant | <0.001 |
| (Table 2) Nails, Age | significant | <0.001 |
| (Table 2) Nails, Sex | significant | <0.001 |
| (Table 2) Nails, Smoking | significant | <0.001 |
| (Table 2) Nails, Alcohol | significant | 0.002 |
| (Table 2) Nails, BMI | significant | <0.001 |
| (Table 2) Nails, Age of onset | significant | <0.001 |
| (Table 2) Nails, Current BSA | significant | <0.001 |
| (Table 2) Nails, Flares | significant | <0.001 |
| (Suppl. Table 6) Scalp vs. Face | significant | <0.001 |
| (Suppl. Table 6) Scalp vs. Palms | significant | <0.001 |
| (Suppl. Table 6) Scalp vs. Soles | significant | <0.001 |
| (Suppl. Table 6) Scalp vs. Genitals | significant | <0.001 |
| (Suppl. Table 6) Scalp vs. Nails | significant | <0.001 |
| (Suppl. Table 6) Face vs. Scalp | significant | <0.001 |
| (Suppl. Table 6) Face vs. Palms | significant | 0.000 |
| (Suppl. Table 6) Face vs. Soles | significant | <0.001 |
| (Suppl. Table 6) Face vs. Genitals | significant | <0.001 |
| (Suppl. Table 6) Face vs. Nails | significant | <0.001 |
| (Suppl. Table 6) Palms vs. Face | significant | 0.000 |
| (Suppl. Table 6) Palms vs. Scalp | significant | <0.001 |
| (Suppl. Table 6) Palms vs. Soles | significant | <0.001 |
| (Suppl. Table 6) Palms vs. Genitals | not significant | 0.742 |
| (Suppl. Table 6) Palms vs. Nails | significant | <0.001 |
| (Suppl. Table 6) Soles vs. Face | significant | <0.001 |
| (Suppl. Table 6) Soles vs. Scalp | significant | <0.001 |
| (Suppl. Table 6) Soles vs. Palms | significant | <0.001 |
| (Suppl. Table 6) Soles vs. Genitals | not significant | 0.419 |
| (Suppl. Table 6) Soles vs. Nails | significant | <0.001 |
| (Suppl. Table 6) Genitals vs. Face | significant | <0.001 |
| (Suppl. Table 6) Genitals vs. Scalp | significant | <0.001 |
| (Suppl. Table 6) Genitals vs. Palms | not significant | 0.750 |
| (Suppl. Table 6) Genitals vs. Soles | not significant | 0.398 |
| (Suppl. Table 6) Genitals vs. Nails | significant | <0.001 |
| (Suppl. Table 6) Nails vs. Face | significant | <0.001 |
| (Suppl. Table 6) Nails vs. Scalp | significant | <0.001 |
| (Suppl. Table 6) Nails vs. Palms | significant | <0.001 |
| (Suppl. Table 6) Nails vs. Soles | significant | <0.001 |
| (Suppl. Table 6) Nails vs. Genitals | significant | <0.001 |

**Supplementary Table 8 – Distribution of psoriasis severity and affection of a hard-to-treat area**

|  | **Mild psoriasis** | **Moderate psoriasis** | **Severe psoriasis** |
| --- | --- | --- | --- |
|  | (0 < BSA < 3) | (3 ≤ BSA < 10) | (BSA ≥ 10) |
|  | (n=1,166) | (n=851) | (n=923) |
|  |  |  |  |
| Scalp | 561 (48.1) | 492 (57.8) | 610 (66.1) |
| Face | 322 (27.6) | 356 (41.8) | 494 (53.5) |
| Palms | 157 (13.5) | 195 (22.9) | 180 (19.5) |
| Soles | 195 (16.7) | 210 (24.7) | 204 (22.1) |
| Genitals | 146 (12.5) | 154 (18.1) | 251 (27.2) |
| Nails | 299 (25.6) | 265 (31.1) | 391 (42.4) |
|  |  |  |  |
| At least one difficult-to-treat area | 937 (80.4) | 747 (87.8) | 821 (89.0) |
|  |  |  |  |
|  |  |  |  |
